# Operational challenges of engaging development partners in district health planning in Tanzania

**DOI:** 10.1186/s12889-022-12520-6

**Published:** 2022-01-29

**Authors:** James C. Kiologwe, Ukio Kusirye, Axel Hoffman, Albino Kalolo

**Affiliations:** 1 Ministry of Health, Community Development, Gender, Elderly and Children, P.O.Box.743, Dodoma, Tanzania; 2 Regional Medical Officer, Morogoro Regional Secretariat, P.O.Box 650, Morogoro, Tanzania; 3 Swiss Tropical and Public Health Institute, Basel, Switzerland; 4 Department of Public Health, St. Francis University College of Health and Allied Sciences, P.O.Box 175, Ifakara, Tanzania

**Keywords:** Stakeholders engagement, Development partners, District health planning

## Abstract

**Background:**

Development Assistance for Health (DAH) represents an important source of health financing in many low and middle-income countries. However, there are few accounts on how priorities funded through DAH are integrated with district health priorities. This study is aimed at understanding the operational challenges of engaging development partners in district health planning in Tanzania.

**Methods:**

This explanatory mixed-methods study was conducted in Kinondoni and Bahi districts, representing urban and rural settings of the country. Data collection took place between November and December 2015. The quantitative tools (mapping checklist, district questionnaire and Development partners (DPs) questionnaire) mapped the DPs and their activities and gauged the strength of DP engagement in district health planning. The qualitative tool, a semi-structured in-depth interview guide administered to 20 key informants (the council health planning team members and the development partners) explained the barriers and facilitators of engagement. Descriptive and thematic analysis was utilized for quantitative and qualitative data analysis respectively.

**Results:**

Eighty-six per cent (85%) of the development partners delivering aid in the studied districts were Non-Governmental Organizations. Twenty percent (20%) of the interventions were HIV/AIDS interventions. We found that only four (4) representing 25 % (25%) DPs had an MOU with the District Council, 56 % (56%) had submitted their plans in writing to be integrated into the 2014/15 CCHP. Six (6) representing 38 % (38%) respondents had received at least one document (guidelines, policies and other planning tools) from the district for them to use in developing their organization activity plans. Eighty-seven point 5 % (87.5%) from Bahi had partial or substantial participation, in the planning process while sixty-two point 5 % (62.5%) from Kinondoni had not participated at all (zero participation). The operational challenges to engagements included differences in planning cycles between the government and donors, uncertainties in funding from the prime donors, lack of transparency, limited skills of district planning teams, technical practicalities on planning tools and processes, inadequate knowledge on planning guidelines among DPs and, poor donor coordination at the district level.

**Conclusions:**

We found low engagement of Development Partners in planning. To be resolved are operational challenges related to differences in planning cycles, articulations and communication of local priorities, donor coordination, and technical skills on planning and stakeholder engagement.

**Supplementary Information:**

The online version contains supplementary material available at 10.1186/s12889-022-12520-6.

## Background

As Low and Middle-Income Countries (LMIC) strive to strengthen their health systems, considerable efforts should be directed towards strengthening the District Health System (DHS). DHS is defined as a decentralized block of the national health system. DHS serves the majority of the population and is a means of achieving an equitable, responsive and people centred health system [1–5]. Provision of comprehensive primary health care services to the population as a core function of the DHS, can be achieved if the DHS is versed in priority setting and planning on the use of the available scarce resources [6–8]. While laying down the DHS concept, the Harare declaration in 1987 put emphasis on the district planning process as one of the core activities of the DHS [9]. Planning using the bottom up approach that ensures participation of a range of actors with a stake on provision of health services in the DHS is a desirable means of reaching the Primary Health Care (PHC) goals and hence paving the way to Universal Health Coverage (UHC) [3, 10, 11]. The participation of state and non-state actors such as Development Partners (DPs), the civil society, philanthropies, private for profit entities and the general public in planning the district health services is a stride forward for provision of equitable, comprehensive, quality and people centred health services [6, 12].

Involving a range of relevant multiple stakeholders in the district planning can increase legitimacy, credibility, transparency and ownership of the DHS plan. Moreover, it can facilitate uptake of the interventions as awareness and mutual trust of the players is maximized through the participatory planning approach [13, 14].. Through stakeholder engagement, a range of important issues for projects success are addressed and may include such issues as cultures, expectations and perceptions [15].

The relevance of participatory planning using a range of stakeholders is highlighted in existing evidence and has been examined in both in High Income Countries (HIC) and LMIC [16] and across a range of health care services [7, 12]. Among the relevant stakeholders in the DHS planning process are the development partners (DPs).

Development Assistance for Health (DAH) in the form of aid has been an important source of health financing in many LMIC [17]. Of recent, the traditional approach of providing aid to “poor” countries by a handful of bilateral or multilateral agencies has been replaced by a more complex way of providing aid to countries with different levels of development and with new players in development assistance. Today stakeholders include bilateral and multilateral agencies, international Non-governmental organizations (NGOs), foundations, global alliances, consortia, philanthropies, initiatives and private corporations [17–21]. As a result, the number of DPs with a stake with district health services has been increasing and diversified.

Although declining in Tanzania, Development Assistance for Health (DAH) in form of aid, including concessional loans, still provides more than 10 % (10%) of the Government budget and a disproportionate share of the financing for development and investment [22, 23]. DAH is provided by the development partners group (DPG) which comprises of seventeen (17) bilateral and five (5) multilateral agencies (counted as one).

In 1999, the health sector in Tanzania entered into a Sector-Wide Approach (SWAP), a government initiative that aimed to improve efficiency in the use of domestic funds and externally sourced development assistance by integrating the funds into a joint sectoral framework [24]. In addition, SWAP aimed to align all forms of Development Assistance for Health (DAH) to address local health priorities and not individual donor priorities in order to avoid fragmentation and duplication of efforts. Through SWAP, the government sets priorities and mobilizes donor support. SWAP in the health sector was developed in response to widespread dissatisfaction with fragmented donor-sponsored projects and prescriptive adjustment lending [25, 26] .It was intended to provide a more coherent way to articulate and manage government-led sectorial policies and expenditure frameworks and build local institutional capacity as well as offer means to more effective relationship between government and donor agencies [27]. In order to properly manage donor support, certain institutional reforms and capacity building had to be undertaken to meet donor requirements. In the Tanzanian context as elsewhere in LMICs, three categories of DAH under SWAP are implemented and include: 1) Budget support: These are donor efforts to support shortfalls in the overall government funding on health in the country. They are not directed to any specific intervention, but to the overall country budget on health in a specific financial year. 2) Health sector Basket Fund (HSBF), mainly a collective effort by donor countries and a recipient government to jointly contribute financially to support health initiatives at the primary health care level on yearly basis. This has and still is the main source of recurrent funding to the districts 3) Projects, these have a specific project life span and address specific areas of interventions. Support can be financial or in kind as we have seen with Tuberculosis, HIV/AIDS, Malaria, and other related health programs.

Development partners in the health sector face several challenges as their counterparts in other sectors in the course of implementing development activities in the recipient countries [28–31]. The challenges range from establishing terms of reference (Memoranda Of Understanding (MOU)) with country governments, governance and coordination of partnerships, misalignment between recipient needs and development partner interest, poor institutional arrangements for public private partnerships (PPPs), power relations to unfavourable political situations. Such challenges when remain unaddressed have profound effects on the performance of both parties in the partnership. The challenges can have effect on several process and functions expected to be performed by the DPs, including the DP participation in health planning processes including the DHS planning.

There is a shortage of information on the operational challenges of involving DPs in the district planning. Understanding such challenges is important in the stride forward to maximize DPs participation at the local level (in this case the district) where donor funded projects (through basket fund or vertical projects) are implemented. DP participation in the district planning process helps to maximize transparency, accountability and trust, which in turn helps to maximize aid effectiveness. Moreover, the process may help to strengthen the district health planning processes as the processes and tools are scrutinized by different stakeholders including the DPs.

Participation of the DPs in the DHS activities could be direct or through the subcontracted (implementing) partners. In Tanzania the district planning is structured. There are clear planning cycles, processes, resource allocation guidelines as well as guidelines of financial and accounting systems. The District Comprehensive Council Health Plan (CCHP) guidelines stipulates the planning cycle, the composition of the planning team, steps in the planning and budgeting processes and evaluation of the plans. The district planning process usually lasts for a period of 5 months between October and March of the financial year. The CCHP planning teams is composed of a range of stakeholders that include the Council Health Management Team (CHMT) members, head of programs or units in the council, representatives from the Regional Health Management Team (RHMT) and non-state actors (including private for profit and non-profit partners such as DPs). The comprehensive council health planning process is conducted at two levels; health facility level and CHMT level, whereby plans from these two levels are later consolidated to a Comprehensive Council Health Plan (CCHP). The planning process usually starts with collecting health priorities from the communities, primarily through the community structures such as; the community health workers, village health committees and health facility-governing committees (HFGC). These are then submitted and consolidated at the health facility level. The health facility Management team prepares a facility plan based on priorities, guidelines, directives and, resource allocation. That plan is then submitted to the Council Health Management Team (CHMT). The CHMT prepares and consolidates the facility plans to prepare a CCHP that after thorough analysis is submitted to the Regional level and if approved to the National levels. In fulfilling the DHS functions which include mixing and allocating resources to improve the health status of the community, the Council Health Management Team (CHMT) is charged with the responsibilities of planning, implementing, monitoring and evaluating health services delivery. In order to fulfil this task successfully, the teams are required to work together and involve different stakeholders at all stages (planning, budgeting and implementing) of their work.

To facilitate smooth planning processes, well-coordinated stakeholder involvement is crucial [8, 32]. The CHMTs are mandated to coordinate stakeholders’ involvement. Utilizing the planning and reporting tool (planrep) [33] a stakeholder’s planning forum is held, with the goal of aligning stakeholders (including the DPs, through their plans and budgets), with district, regional and national priorities thereby integrating them into the CCHP. This process ensures that all efforts from various sources are directed towards addressing local, regional and national health priorities. The activities and contribution of each stakeholder are monitored and accounted for, preventing a multiplicity of efforts and chaos. Tools such as Health Management Information System (HMIS), research reports, district financial and, technical reports are utilized in the planning process assuring adherence to government mandated planning cycle and related guidelines. When there are inadequacies of the tools or poor communication about the availability of such tools to the planning team including the DPs, the planning process may lead to delays and inefficiencies. Aware of this health officers at all levels are trained in employing such tools assiduously.

The aim of the current study is to examine the engagement of Development Partners, specifically their subcontracted implementing partners, in the district health planning in Tanzania. Engagement is hereby defined as a process of involving individuals and groups that either affect or are affected by the activities of the organisation [34]. Our goals are to 1) identify the DPs involved in the CCHP planning process and how they are engaged 2) determine their perceptions and awareness of the CCHP planning process and 3) identify operational challenges affecting the engagement of the DPs in the CCHP planning process.

## Methods

### Study settings

This study was conducted in Bahi and Kinondoni districts of Tanzania Mainland between November and December 2015. Kinondoni is an urban district (Municipality) in the Dar es Salaam Region, the business capital of Tanzania Mainland. It has a total area of 321 km^2^ and population of about 1,134,211 people [35] . Bahi is one of seven rural districts in the Dodoma Region. Located fifty (50) kilometres from Dodoma town and five hundred (500) kilometres from Dar es Salaam, it has a population of 221,645 [35] . The study was conducted in both urban and rural districts for the purpose of comparing urban and rural districts in the CCHP development in relation to also engaging the DPs. Kinondoni was purposively chosen to represent urban districts due to its location in Dar es Salaam — Tanzania’s largest city and its business and financial hub. Because of this, DPs are more concentrated in Dar es Salaam than other regions. On its part, Bahi District was selected purposively to represent rural districts.

### Study design

This is a mixed methods case study that employs both qualitative and quantitative approaches in data collection and analysis. In this study, we followed the Good Reporting of A Mixed Methods Study (GRAMMS) framework for reporting our findings [36]. Taking a pragmatic stance, the mixed methods approach was set to capture information related to DP engagement in district planning process from multiple perspectives and provide an in-depth understanding of the process in the natural context. This study was explanatory in nature. We started with the quantitative approach and followed with qualitative interviews for selected items to give details on the observations obtained in the quantitative strand. The quantitative strand of the study involved the following activities; i) mapping of the DPs and their activities in each district, ii) assessment of the level of engagement of the DPs and district planning teams in the planning process and, iii) determining perceptions and awareness of the CCHP planning process. The qualitative part describes how the DPs engage in the district health planning processes and reveals the challenges of the process as described by the participants.

### Study sample and sampling procedures

The cohorts in this study are 1) The Council health planning team (CHPT) members and 2) The Development Partners (DPs), here defined as organizations/agencies engaging with health development assistance at the local government (district) level, including multilateral/bilateral district projects, and NGOs (international, national, and regional) operating in the particular district.

The quantitative information was collected using 1) A mapping checklist for DPs which was completed by a CHPT member in each district 2) A district assessment questionnaire that was completed by a CHPT member in each district 2) DPs assessment questionnaire, completed by sixteen (16) DPs.

The DPs mapping checklist was completed by the CHPT secretary (i.e., the district health secretary) and the district assessment checklist was completed by ten (10) purposively selected CHPT members (including the Regional health management team member who serves as an advisor to the CHPT). The sixteen (16) DPs chosen to complete the questionnaire were selected using a stratified sampling technique based on district type, type of partner, area of support, and status of submission of partner plans to the district. Although the mapping tool recorded a total of thirty-five (35) DPs, some were excluded to participate in the survey because they were either inactive for more than a year, or were represented by other DPs who implement similar interventions, or receive funding from similar prime donors.

The qualitative sample included twenty (20) key informants who participated in in-depth interviews. The participants were selected purposively and include the DPs (*n* = 8), members of the CHPT (*n* = 10) and regional level officials who support CCHP planning (*n* = 2). See Table 1 for information regarding the sample.

**Table 1:** Distribution of Sampled study participant

Sample Descriptions/subjects	Sample Size
Preliminary assessment of DPs	35
Quantitative assessment	
Key respondents to specific questions (From each district)	2
Development Partners	16
**Total for Quantitative assessment**	**18**
**In-depth Interview**	
CHPT (Five From each district -DMO, DHS, DRCHCO, DCDO, DPLO)	10
Development partners	8
Regional Health Management Team officials (RMO/RHS and PPP Coordinator	2
**Total IDI**	**20**

### Data collection tools and procedures

Data for both the quantitative and qualitative strands of the study were collected by trained research assistants and were supervised by J.K. Data collection tools were pretested before actual data collection.

### Collection of quantitative data

We used the following tools to collect quantitative data; i) A mapping checklist ii) District Assessment Questionnaire iii) DPs Assessment questionnaire.

#### A mapping checklist

We used a structured checklist to map the DPs in the district. The information collected included: name of the DP, type of the DP (multilateral, bilateral, NGO (international, national, Regional, District), area of support, duration of operation in the district, and their physical and contact address. (See Additional file 1).

#### District assessment questionnaire

The district assessment questionnaire collected information related to: number of DPs who have an Memorandum of Understanding (MOU) with the council, DPs who have submitted their plans in writings to be integrated into the 2014/15 CCHP, how the district priorities are communicated to DPs, perceived reason for the engagement or non-engagement of DPs in CCHP, perceived level of participation of DPs generally and to the specific CCHP planning processes and how the implementation of DP activities is coordinated and monitored. Additional file 2 provides more details.

#### DPs assessment questionnaire

The DPs assessment questionnaire was used to measure; the perceived understanding of DPs on the CCHP Planning processes, communication strategies and approaches between DPs and the District, District priorities and how they are set, level of engagement of DPs to CCHP, reasons for DPs to engage or not engage in CCHP planning and, barriers and facilitators of engagement in the CCHP planning process. Development partners engagement in the planning process were assessed by; determining whether they had a Memorandum Of Understanding (MOU) with the council, submitted their plans to the council for CCHP planning and received communication from the council on materials for planning (guidelines and policy documents) and, timelines of the planning session, including the sessions they are invited to present their plans. In addition, DPs were asked to gauge their own participation in CCHP planning process. Additional file 3 provides more details.

### Collection of qualitative data

Qualitative data were collected using a semi structured in-depth interview guide (see Additional file 4 a & 4b). The guide collected information related to understanding the rationale of stakeholder involvement in CCHP, Processes of preparing the CCHP, reasons for participation or non-participation in the CCHP planning process. In addition, soliciting for information on if there is any situation that restricts the district from involving DPs in the preparation processes of the CCHP, effects of non-engagement of DPs, causes and mechanism to ensure compliances.

### Data analysis

Guided by the research objectives, the collected data were analysed using descriptive statistical approaches and a thematic analysis to obtain quantitative and qualitative findings respectively.

### Quantitative data analysis

Data from the questionnaire were analysed descriptively to generate frequencies percentages and means on the key variables of the study namely; 1) participation in comprehensive council health plan, measured as scores and categorized into full participation, partial participation and no (zero) participation 2) presence of MOUs between the DP and the district, DP, 3) submission of DPs activities in writing for inclusion in CCHP, 4) Pre-planning meeting, feedback on finalization of CCHP and number of DP participating in quarterly CCHP monitoring meeting. SPSS version 18 for Windows (SPSS Inc., Chicago, IL, USA) was used in data cleaning and analysis.

### Qualitative data analysis

Qualitative data was transcribed verbatim and thematic analysis approach was used to obtain the themes including their related codes and categories. All steps from transcription, coding, identification of categories and themes followed specific recommendations from the literature on qualitative data analysis [37–39] Data was analysed using the following steps; 1) Familiarizing ourselves with the data by reading and rereading the transcripts 2) Organizing our data in a meaningful and systematic way by generating codes 3) Organizing the codes into themes 4) Reviewing the themes to see if they are actually related to our data and research questions 5) Refining the themes in order to identify the ‘essence’ of what each theme is about. The analysis was mainly explanatory and we used an abductive approach (a mixture of deductive and inductive) in coding the data. Although we were guided by our research questions, we allowed for themes emerging from the collected data (as in grounded theory). In the entire process, we took a programmatic stance [40, 41]. To ensure rigor, a combination of field notes and transcripts from audio clips were used during coding and the transcripts were read by at least two of the authors. Furthermore, the findings were triangulated across the two groups of participants, and we use the quotes from the participants in reporting our findings.

## Results

### Characteristics of study participants

Information for this study was collected from a total of sixteen (16) DPs but also from district officials and RHMT representatives. Seventy-five percent (75%) of the DPs who participated in the survey were NGOs (both international and national). Bilateral projects, multilateral projects and Faith Based Organisations (FBOs) also provided information for this study. FBOs were predominantly found in the rural district. Sixty-nine percent (69%) of the DPs had supported their respective districts for more than five (5) years.

Participants in in-depth interviews comprised of 50 % (50%) males and females. Most of the participants were DPs. See Table 2 for further information on study participants.

**Table 2:** Characteristics Of Study Participants

Type of partner’s organization	Rural	Urban	Total
International NGO	2	4	6
National NGO	3	3	6
Bilateral project	1	0	1
Multilateral project	0	1	1
FBO	2	0	2
**Life span of the projects supported by DPs**			
≤ 5 years	2	3	5
> 5 years	6	5	11
**In-depth interview participants by sex**			
Male	4	6	10
Female	5	5	10
**In-depth interview participants by position**	9	11	20
District officials	4	4	8
DP Representative	4	6	10
RHMT	1	1	2

### Distribution of development partners and supported interventions

**Fig. 1:**
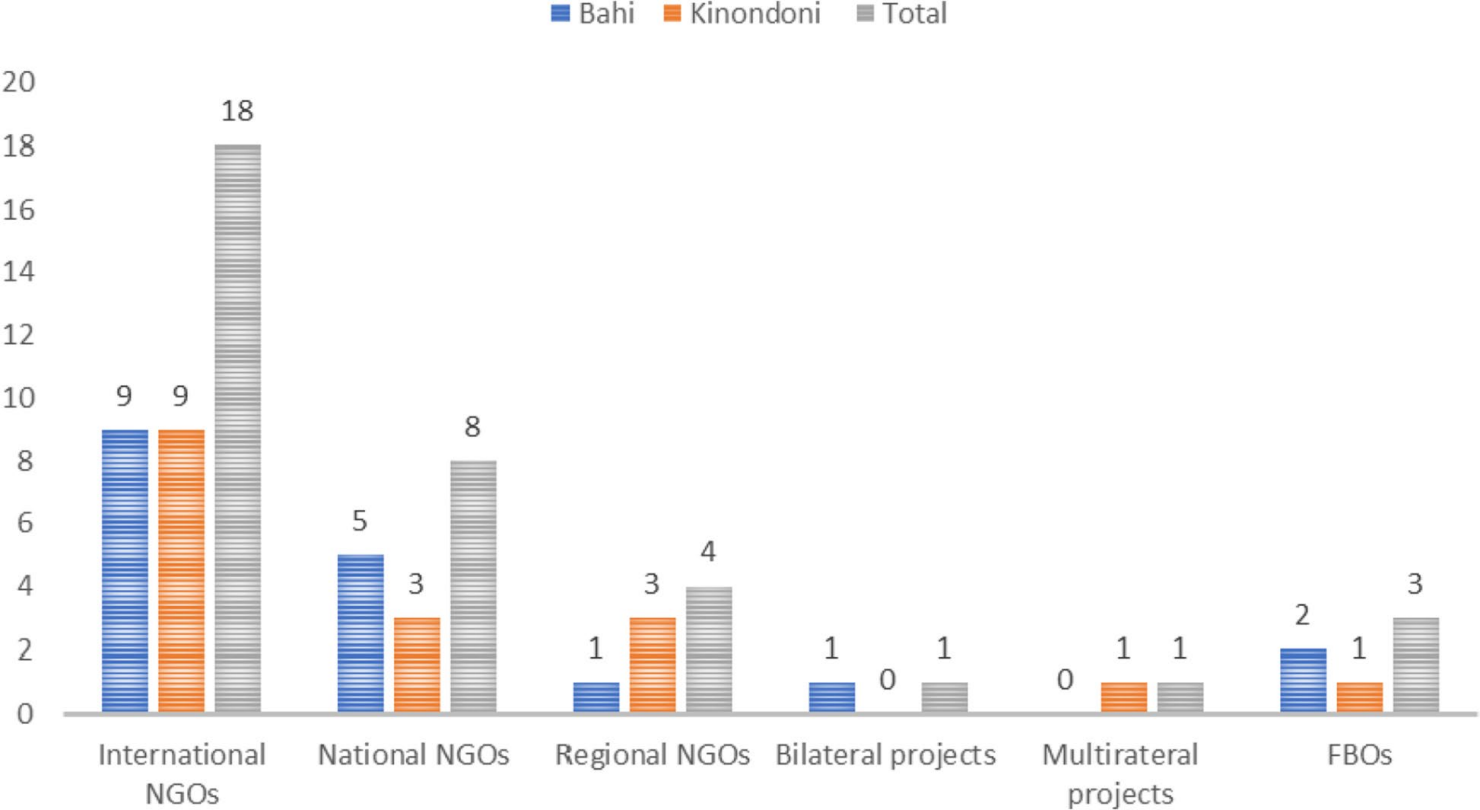
Type Of Development Partners (DPs) By District

Our mapping tool identified a total of thirty-five (35) eighteen (18) from Bahi and seventeen (17) from Kinondoni) development partners (excluding national programmes) supporting the health sector in the two districts (see Fig. 1). Non-governmental organisations (NGOs) both international, national and those at the regional level (thirty or 85 % (85%) of all DPs, were the most predominant DPs in the districts. We found no difference in number of DPs between the two studied districts.

We found that HIV/AIDS (20 % (20%)), Food and Nutrition (15 % (15%), Family Planning (14 % (14%)) Reproductive Maternal Neonatal and Child Health (RMNCH) (twelve perecent (12%) and Water Sanitation and Hygiene (WASH) (10 % (10%)) were the common health interventions receiving support from partners as shown in Table 3 below. Some of the partners were supporting more than one intervention in the district. In addition, Bahi district received more interventions (58 % (58%)) than Kinondoni.

**Table 3:** Types Of Health Interventions Supported By DPs Per District

Type of intervention	Bahi	Kinondoni	Total number of interventions (%)
Capacity building to CHMT	0	3	**3 (7)**
Capacity building to health care workers	1	1	**2 (5)**
Support to the Community health Fund (CHF)	2	0	**2 (5)**
HIV/AIDS interventions	4	4	**8 (20)**
WASH interventions	3	1	**4 (10)**
Food and Nutrition	6	0	**6 (15)**
Support to Most Vulnerable Children (MVC)	2	0	**2 (5)**
Family planning (FP)	2	4	**6 (14)**
Reproductive Maternal Neonatal and Child Health (RMNCH)	2	3	**5 (12)**
Equipment and construction of infrastructure	2	1	**3 (7)**
**Total number of interventions per district**	**24**	**17**	41 **100)**

### Engagement of the DPs in the CCHP planning process

We found that only four (4) or 25 % (25%) of DPs had an MOU with the council, 56 % (56%) had submitted their plans in writing to be integrated into the 2014/15 CCHP. Six (6) or 38 % (38%) respondents reported to have received at least one document (guidelines, policies and other planning tools) from the district for them to use in developing their organization activity plans. CCHP template and guidelines were the commonest documents shared; none reported to have been using district health strategic plan and annual District CCHP in developing their plan. Bahi district performed better in all aspects than Kinondoni. Review of the CCHP further showed that only 8 % (8%) of the DP activities submitted were included in the CCHP. More information is found in Table 4.

**Table 4:** Engagement In The Council Planning Process

Engagement in the Council Planning activities	Bahi	Kinondoni	Total
Have signed MOU with the council	3	1	4
Have submitted activities for inclusion in CCHP	6	3	9
Received communication from the district on priorities of the CCHP in the respective year	3	3	6

Respondents in qualitative interview indicated that DPs take part in planning process and highlighted that they submit their written plan and they are invited in planning workshops.

*“Yes, we share our plans in written form. If it happens that more than one DP organisations are implementing similar activities, we discuss and agree among each other who should continue and who should change the intervention or the implementation sites.” (IDI-district development partner 2, Bahi).*

*“We are often invited to participate in a planning meeting together with the CHPT whereby each of the DPs presents the area of support and in fact reports what has been implemented in the last year, if necessary and what to be implemented in the next planning cycle.” (IDI- district official 2, Kinondoni).*

### DPs’ participation to specific areas of CCHP planning process

The Participants in the DPs survey were asked to gauge their organization level of participation in the process of developing their respective district annual health plan (CCHP) as a general view and to specific CCHP development process. Eighty-seven point 5 % (87.5%) of the respondents from Bahi had partial or substantial participation, while sixty-two point 5 % (62.5%) of respondents from Kinondoni had not participated at all (zero participation). (See Table 5).

**Table 5:** DPs’ Participation To Specific Areas Of CCHP Planning Process (*N* = 16)

Stage	Gauge	Rural	Urban	Total
Identifying priority health problems /intervention to be addressed in the 2014/15 CCHP Plan	Zero participation	1	4	5
	Partial Participation	4	2	6
	Full Participation	3	2	5
Allocating resources to the interventions	Zero participation	1	4	5
	Partial Participation	5	2	7
	Full Participation	2	2	4
Developing CCHP Action Plan	Zero participation	0	5	5
	Partial Participation	5	1	6
	Full Participation	3	2	5
Developing the capacity of the Council Health Planning Team	Zero participation	5	5	10
	Partial Participation	3	1	4
	Full Participation	0	2	2
Implementation of CCHP activities	Zero participation	0	5	5
	Partial Participation	3	1	4
	Full Participation	5	2	7
Evaluation and quarterly reporting	Zero participation	1	5	6
	Partial Participation	3	2	5
	Full Participation	4	1	5

### DPs’ perceptions and awareness on the planning processes and benefits of integrating their plans into CCHP

We found that sixty-two point 5 % (62.5%) of the DPs from Bahi district perceived the district health planning team capacity to do the planning as excellent compared to twelve point 5 % (12.5%) from Kinondoni district. It was revealed that eighty-seven point 5 % (87.5%) of the DPs from Kinondoni district perceived the process as average, low or poor.

**Fig. 2:**
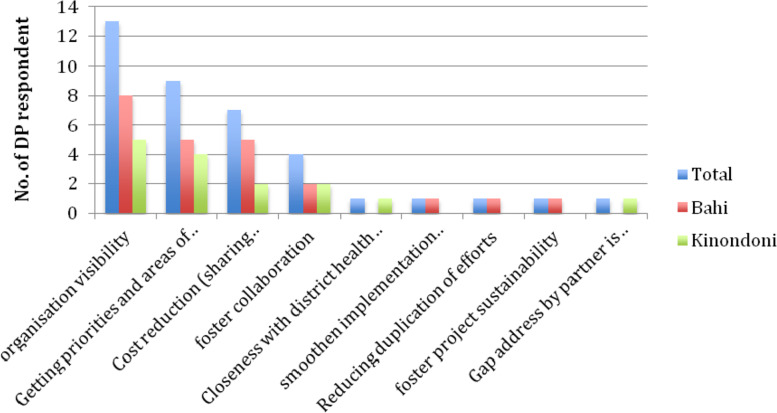
DPs’ Perceived Benefits Of Engaging In CCHP

DPs were aware of the benefits for DPs to participate and integrate their activities into the district CCHP. Some of the mentioned benefits were: organizational visibility, recognition of the importance and presence of the organization by the district and, securing priorities and areas of working and reducing running costs of an institution due to resources sharing (see Fig. 2).

### Operational challenges affecting the engagement of the DPs in the CCHP planning process

We found a range of operational challenges to partners’ engagement in the CCHP planning process. Participants in the DPs survey pointed to the following challenges; being not aware of the guidelines and government regulations, procedures and policies for planning and integration of partner plans (Mean 3.125, SD = 1.6421), absence of transparent/clear procedures from the district management to development partner (Mean = 3.250, SD = 1.4311) and being not directed by prime donor/funder (Means = 3.1250, SD = 1.8871). Other reported reasons were differences in financial year (planning cycle) between various partners and the government, delay/no assurance of grants from the prime donors, being given short notice to submit their plans to Districts.

Participants in the key informant interviews reported various challenges deterring the DPs from engaging in CCHP. These challenges were both from partners and the government. The identified challenges are presented here below with their supporting quotes from the participants.

#### Differences in budget timeline

Participants reported differences in budget timelines between the government and that of donors as a stumbling block in engaging the partners in planning specifically getting their pledges in supporting CCHP activities

*“There is a problem of budget timeline. Whereas our financial year ends in June, the financial year of most of our partners start in October. This means that, by the time you engage them, they might say ‘we will see what to do’ but no commitment. Given these financial year differences, it becomes difficult to submit a paper of what they want to do because, and their donation would then not have been confirmed by their funder.” (IDI-district official 1, Kinondoni).*

#### Low predictability of funding from prime donors

The development partners who receive funding from prime donors sometimes face uncertainties of whether their proposed projects will be accepted or not as their funding is subject to competitive bidding processes

*“One NGO which had never submitted its plan and we even have had not seen them in the field, came to our office asking us to rate them well as an independent assessor was coming for midterm review of the project.”. (IDI- district official 3, Bahi).*

#### Inadequate financial allocation for planning activities

Some DPs reported that they often do not set aside funds for CCHP planning activities. The DPs are expected to participate in CCHP planning and bear most of the costs using their own funds. If they have not set aside some funds for this activity, it becomes difficult to participate.

“*There is a serious challenge on our side as we often don’t have the budget to enable our staff to attend, let’s say a five day CCHP planning which is usually done outside their district. So we end up by presenting our action plan to CHMT for them to consolidate. However, we do not get feedback from them whether they have included it or not.” (IDI-district development partner 2, Bahi).*

*“When we invite development partners into the planning, we do not pay them, so those who can pay for themselves we normally accept them and we cooperate with them. Hence, we do not have cost implication except for meal and refreshments that we can accommodate.” (IDI-district official 2, Kinondoni).*

### Few /irregular meetings

Some participants reported of few meetings organized by the districts where DPs are invited. This is one of the serious challenges that hinder DPs participation in CCHP planning sessions or submitting their plans to be included in the CCHP.

*“I have been here for five years and have never been invited even to a single district coordination meeting.” (IDI-development partner 3, Bahi).*

*“We have in our council meeting schedule a quarterly NGO coordination meeting, but we often don’t hold these meetings as scheduled due to inadequate financing.” (IDI- district official 2, Kinondoni).*

### Lack of transparency

It was also reported that some DPs are not transparent on what they have at their disposal to be included in the CCHP or to support the district.

*“Most development partners are not transparent on their budget. They also never tell you their future commitments to the district. For example, that ‘we have this amount of money and we want to do this and that in this area of health.” (IDI- district official 1, Bahi.)*

### Limited knowledge and skills amongst CHPT on planning

Knowledge and skills to engage partners and coordinate well the CCHP planning was reported as a limitation. The CHPT may need more capacity building on this aspect, as it has been a challenge in the studied districts.

*“Developing a comprehensive council health plan is a technical activity requiring people who are knowledgeable and skilled in planning health-related activities. However, our staffs have not been well exposed to such type Of Trainings.” (IDI-District Official 3, Bahi).*

### Lack of sufficient and technically qualified workforce in LGAs

Participants in interviews reported that the lack of human resources in LGAs hampers the efficiency in the CCHP planning process.

*“Potential employees, especially recently graduated young ones, do not like to work in the rural areas where there is poor working environment, particularly lack of staff houses, electricity, good office facilities and poor transport.” (IDI district official 1, Bahi).*

### Limitations of the planning tool (plan rep)

The planning tool (Plan rep) was reported by the respondents that it has some challenges in accommodating partners plans especially if there are some delays from genuine reasons such as delays in approval of their activities from prime donors.

*“The planrep is not flexible to add our partners who do not appear in the planrep version. Therefore, we end up having difficulties entering their plans into planrep. Even in the quarterly reports, we do not report their activities.” (IDI district official 2, Bahi).*

**Fig. 3:**
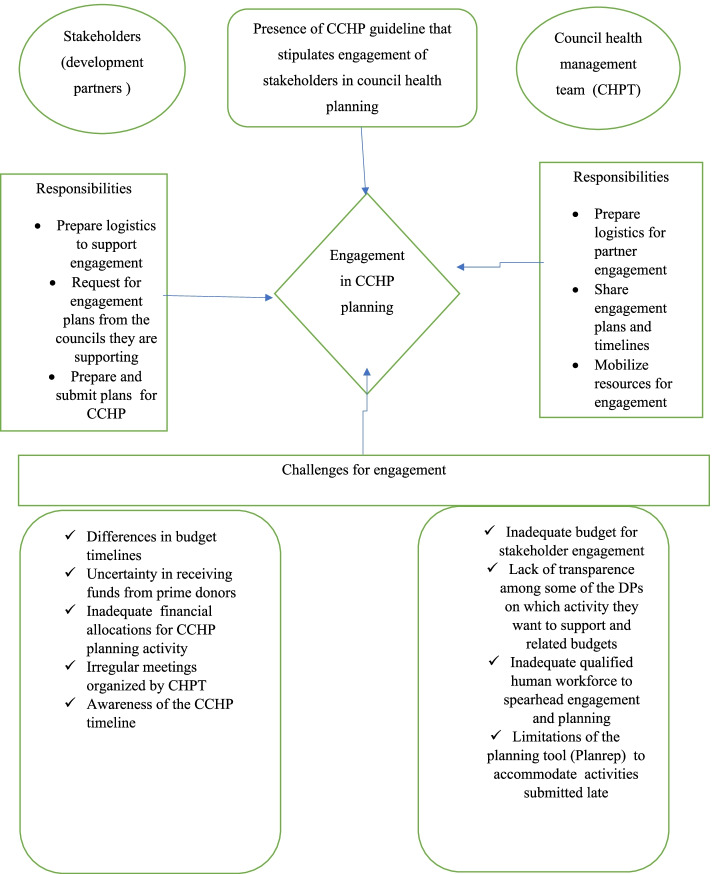
A Summarized Model For Partner Engagement In CCHP

One hundred percent (100%) of participants in interviews stated that a successful partner engagement is possible if there are clear guidelines for engagement and the responsibilities of each part are present and well understood by all parties. Figure 3 provides a summarized model for partner engagement in CCHP in the current study and displays the structures for engagement, responsibilities of each part in the engagement and the challenges for engagement.

## Discussion

This study examines the engagement of health development partners (DPs) with the district health planning in two districts, representing rural and urban settings in Tanzania. Generally, the findings suggest low participation of DPs in the health planning process at all stages of comprehensive council health planning, with worse situation in urban settings. Important factors explaining this situation include; differences in planning cycles between the government and donors, uncertainties in funding from the prime donors, lack of transparency, limited skills of district planning teams on stakeholder involvement and, technical practicalities related to planning tools and processes.

The reported low-engagement of development partners in the planning process is a major concern that may have different implications in the quality of Council Health Plans of the respective districts. Four major issues were identified. 1), the CCHP is expected to include all financial and non-financial contributions of all actors with a stake in the district health system, in the case where the DPs do not participate, the comprehensiveness of the plan is jeopardized. 2), the budgetary transparency of both the DPs and the government can’t be realized since there will be a problem in accessing DPs budgets for activities committed to be implemented in the districts. 3) the CCHP planning is expected to address donor fragmentation and therefore harmonizing their support in a given district, with low participation, it means continuing fragmentation of DPs support to the district health system. 4), the power relations that exist between the DPs and the (local) council governments may present a challenge in ensuring that the two parts are accountable in relation to planning and executing their plans in the council.

Existing evidence attests to the divergent interests; problems of accountability, transparency and problems of power relations as reasons for low engagement and continued fragmentation of aid for DAH and global health initiatives [42, 43].. Efforts to maximize DPs engagement may include such actions as preplanning stakeholder forums that aim at harmonizing DPs support, thus understanding who does what, where and the amount of funds to be allocated for each of the supported activities,

The finding that only few DPs received planning guidance and district priorities to guide planning activities highlights a communication problem between the district planning teams and the DPs. Existing evidence attests to the importance of effective communication that takes into account the multiplicity and complexity of stakeholders [15]. There are accounts of absence of genuine communication or consultation between different actors involved in the district planning process as found in this study [44]. Bonnenberger and colleagues point to inadequate planning and communication skills among district managers [45]. This finding has implication on the participation levels in planning activities and quality of activities included in the plans. Improving communication and coordination through decision support systems and networking among partners working in the same district could help to resolve this problem [46].

We observed a discrepancy in the DPs participation in the planning process between rural and urban settings. The rural based DPs performed better, which is contrary to our initial assumptions that the urban districts have better planning and coordination capacity than the rural district due to presence of competent human resources [47].. This could be explained by easier visibility of partners in rural settings than in their urban counterparts given the multiplicity of stakeholders working with the district health departments in the urban areas.

The finding that the DPs were aware of the benefits of their involvement in the planning process but did not actually participate in the planning processes is not new and could be explained by the fact that awareness and practice are usually not close if there are barriers of the two parties to actually meet. In our study the discrepancy could be explained by the observed operational challenges such as differences in planning cycles between the government and donors, uncertainties in funding from prime donors, lack of transparency, limited skills of district planning teams on stakeholder involvement and, technical practicalities related to planning tools and processes. In their paper, Moon and Omole, allude to the volatility and uncertainty of financing, coordination, priority setting and accountability as a serious limitation of DAH [48].

The inadequate dissemination of national policies and guidelines used for planning observed in this study challenges the role of the Regional Health Management Teams (RHMTs) in supporting districts to develop their CCHP and in disseminating various health policies at the Regional and District level. Furthermore, the observation that even when the District DPs submitted their plans to be included in the CCHP; less was reported as actual expenditure from the council health accounts during the quarterly CCHP reports, highlights a discrepancy between the plan and actual expenditure of DPs activities in the studied districts and raises several questions related to what is planned and what is actually implemented and the reasons behind this discrepancy. Future studies should explore this gap.

Our findings indicate challenges that are to be resolved on the side of the DPs as well as the council health planning teams so that each part fulfils their responsibilities in the planning process and hence providing quality services to the communities. In their study, Frumence and colleagues has called for DPs and CHPTs to work together during the planning stage of the CCHP to ensure that all development partner supported activities are incorporated in the CCHP [28]. Establishing a common ground to resolve this through stakeholder forums and avoiding blame shifting is a stride forward to effective engagement and comprehensive planning in the DHS.

Although data collection took place 6 years ago, with exception of the COVID-19 pandemic, there are few incremental changes to the district health system landscape. The updates to the planning (CCHP) guidelines, and the introduction of the web based planning and reporting (Planrep) tool to accelerate the gains of the direct health facility financing (DHFF) mechanism that is currently being implemented in the primary public health facilities, are the changes that need to be acknowledged [49]. Given the fact that health systems are complex and adaptive, we acknowledge that the COVID-19 pandemic and the incremental changes in the planning processes may cause significant effects in the health system outputs. The fact that there is a dearth in literature in development partners engagement in district health planning, we think that this analysis is still relevant today and has the potential to inform researchers, policy makers and development partners in Tanzania and beyond on modalities to engage in district planning.

### Methodological considerations

In this study we use an explanatory mixed methods approach to provide a comprehensive picture of the DPs engagement in the planning process and the operational challenges of DPs’ involvement in district health planning in Tanzania. The inclusion of a rural and an urban district in a single study helps to uncover the differences and similarities in the two settings. As health planning in Tanzania is further devolving to the health facility level, the study casts light on issues that need to be resolved to enhance partners participation in health planning at various levels of the DHS.

Although in this study we compared two districts, we cannot claim generalisability of the results given the limitations of case study design. However, the reader could identify findings that can be transferable beyond the study area.

## Conclusions

DPs were engaged in District health planning; however, the level of engagement was low and varied greatly between the two districts with Kinondoni (urban district) having lower scores of DPs engagement at all stages of planning. Local government challenges included, low planning knowledge and skills among CHPT, weak oversight of bodies tasked with approving CCHP, reviewing DPs plans and following implementation of DPs plans; weak consultative structures and coordination mechanisms; low negotiation capacity; lack of clear and adequate communication about local government priorities and inadequate financing to the planning process. Central government challenges included, inadequate dissemination of policies, interference with the autonomy of local governments in planning, weaknesses in supporting districts, featured by inadequate tools for monitoring compliance and lack of clear guidance on planning policies and guidelines to DPs. District DPs challenges included lack of formal leadership to coordinate and network DPs in the district, inadequate knowledge of DPs on government policies and guidelines on planning, heavy reliance on external aid for NGOs resulting in low predictability of funding and interventions being skewed towards donor priorities as well as flagship of donor projects thus making it harder to jointly support the sector.

## Supplementary Information

**Additional file 1.**

**Additional file 2.**

**Additional file 3.**

**Additional file 4.**

## Data Availability

All data underlying the findings are fully available without restriction from the corresponding author of this study.

## Abbreviations

CCHP: Comprehensive council Health Plan
CHMT: Council Health Management Team
CHPT: Council health planning Team
DAH: Development Assistance for Health
DHS: District Health system
DP: Development partner
DPG: Development Partners group
IDI: In-depth Interviews
LMIC: Low and Middle income Countries
MOHCDGEC: Ministry of Health, Community Development, Gender, Elderly and Children
NGO: Non-governmental organization
RHMT: Regional Health Management Team
SWAP: Sector wide Approach
UHC: Universal health coverage
