# Supplementary material for: Operational challenges of engaging development partners in district health planning in Tanzania
Source: BMC Public Health. 2022 Jan 29;22:200. doi: 10.1186/s12889-022-12520-6 (PMC8800550; doi:10.1186/s12889-022-12520-6)
Supplement: Supplementary file 1 — Additional file 1. [file 12889_2022_12520_MOESM1_ESM.pdf]

Additional file 1: District Development partner mapping sheet

Name of the DISTRICT…………………………………………………………….

SN	Name of the Health Donor organization	Type of the organization (NGO, FBO. CSO, Donor Project, Other)	Main area of support	Duration in the district	Physical address	Contact person	Mobile number of the contact person
