# Supplementary material for: Operational challenges of engaging development partners in district health planning in Tanzania
Source: BMC Public Health. 2022 Jan 29;22:200. doi: 10.1186/s12889-022-12520-6 (PMC8800550; doi:10.1186/s12889-022-12520-6)
Supplement: Supplementary file 2 — Additional file 2. [file 12889_2022_12520_MOESM2_ESM.pdf]

Additional file 1: District Assessment Questionnaire

PART A: INTRODUCTION

Questionnaire ID: [][ ] Interviewer ID ……….

Date of Interview: dd/mm/yyyy: [][ ] /[ ][ ] /[ ][ ] [ ][ ]

No	Question	Answer	Code
1.	District name	1. Bahi 2. Kinondoni	
2.	Name of interviewee (Optional)		
3.	Position of Interviewee	1. DMO 2.DHS<br>3.DPLO<br>4.NGO representatives<br>5.FBO representative<br>6. DCDO 7. PPP Coordinator	
4.	How long have you been in this position?	[ ][ ] Years	
5.	How many development partners are collaborating with your District? *Please indicate the number		
	a)International NGO	[ ][ ]	
	b)National NGO	[ ][ ]	
	c)Regional NGO	[ ][ ]	
	d)District NGO	[ ][ ]	
	e)Bilateral Projects	[ ][ ]	
	f)Multilateral Projects	[ ][ ]	
	g)Faith Based Organisation (FBO)	[ ][ ]	

	(International/National)		
	h) Faith Based Organisation (FBO)<br>(Regional/District	[ ][ ]	
	i)Total Number of Development<br>Partners	[ ] [ ]	
6.	How many partners have specifically signed MOU with in this District ? ………..Number of MOU		[ ][ ]
7.	The number of partners with MOU duration		
	a)One Year [ ][ ]	b)Two Years [ ][ ]	
	c) 3-5 years [ ][ ]	d) More than 5 Years [ ][ ]	
8.	How many MOU have specific conditions for		
	a) Partner plans development processes?		[ ][ ]
	b) Partner participation in District planning?		[ ][ ]
	c) Coordination Mechanisms?		[ ][ ]
	d) Partner Reporting?		[ ][ ]

	f) Others (Mention)		
11.	Do you usually share these documents with the local DPs?	1. Yes<br>2. No	
12.	If the answer is No to Question Number 11 above, can you explain why?		
	a) We did not have enough copies	1. Yes<br>2. No	
	b) Didn't see the importance of distributing the documents to DPs	1. Yes<br>2. No	
	c) There are no specific directives /not guided to distribute the documents to DPs	1. Yes<br>2. No	
	d) Others (Mention)		
13.	a) Do you have a District Health Strategic Plan?	1. Yes<br>2. No	
	b) If the answer is yes to Question 13a, how many DPs were involved in the preparation of the strategic plan? [][]		
14.	If the answer to Question 13a is Yes, Please explain how the District Development Partners were involved		
	a) In situation analysis /need assessment	1. Yes<br>2. No	
	b) In developing District Priorities	1. Yes<br>2. No	
	c) In consolidation of the plan	1. Yes<br>2. No	
	d) In strategizing funding mechanism for the Strategic Plan	1. Yes<br>2. No	

	e) In coordinating and communicating with other Partners	1. Yes<br>2. No	
	f) Others (please explain)		
15.	If the answer is No to Question 13a, please give the reasons		
	a) Did not know how to develop Strategic Plan but it was important	1. Yes<br>2. No	
	b) Shortage of resources (funds and human resources for developing Strategic Plan	1. Yes<br>2. No	
	c) Not aware of the importance of the strategic plan	1. Yes<br>2. No	
	d) Others (please explain)		
16.	How many DPs have sent their commitments in writings for their plans to be integrated into the 2014/15 CCHP? [ ][ ]		
17.	How many partners' plans have been included into the CCHP for the FY 2014/15? [ ][ ]		
18.	Are there any donor partner plans (commitments) which were submitted in writing but were unable to be included into the district plans (CCHP)?	1. Yes<br>2. No	
19.	If yes to question 18, what is the per cent of unintegrated plans?		
20.	If the answer is yes to question 18, what were the reasons?		
	a) Shortcoming of the planning tools	1. Yes<br>2. No	
	b) Plans were submitted late	1. Yes<br>2. No	
	c) The plans were incomplete /shortcoming of the DP	1. Yes	

	Plans	2. No	
	d) Others please explain		

PART C: PLEASE FILL THE TABLE TO INDICATE PERCEIVED LEVEL OF ENGAGEMENT OF DPS IN PREPARTION OF DISTRICT CCHP

S/N	Question	Level of participation/engagement<br>1. ZERO Participation<br>2. Minimum /Partial Participation<br>3. Substantial /Full Participation
21.	Generally, what is the level of participation of the local Development Partners in CCHP planning?	
22.	What is the level of participation of the district Development partners in the following CCHP Planning Processes:	
	a) Identifying priority health problems /intervention to be addressed in the 2014/15 CCHP Plan?	1. Yes 2. No
	b) Allocating resources to the interventions?	1. Yes 2. No
	c) Developing quarterly/monthly joint Action Plans?	1. Yes 2. No
	d) Developing the capacity of the Council Health Planning Team?	1. Yes 2. No

	e) Implementation of CCHP activities?	1. Yes 2. No
	f) Quarterly reporting and joint monitoring?	1. Yes 2. No

PART D: PERCEIVED REASONS FOR INTERGRATION OR NON-INTERGRATION OF DPs PLANS IN THE DISTRICT CCHP

S/N	Question	Answers	Code
23.	For those DPs who integrated their plans into the District CCHP, What were the perceived benefits for integrating DP plans into CCHP?		
	a) Compliance with government procedures/procedures	1. Yes 2. No	
	b) Directives from the prime donor	1. Yes 2. No	
	c) Reduction of DPs implementation costs	1. Yes 2. No	
	d) Visibility of DP contribution	1. Yes 2. No	
	e) Others (Mention)		
24.	In your opinion, how efficient has the district been in integrating partner plans into the CCHP?	1.Very efficient<br>2.Efficient<br>3.Low efficiency<br>4.Non efficient	

25.	Would you please give explanation for the above answer		
	a)		
	b)		
	c)		
26.	Do you think there are any effects of non-integration of partner plans into CCHP to district performance?	1. Yes<br>2. No	
27.	If yes, what are the effects?		
	a) Wastage of resources due to duplication of efforts	1. Yes<br>2. No	
	b) Over burdening health staffs causing inefficiency in use of human resource	1. Yes<br>2. No	
	c) Multiple uncoordinated reporting	1. Yes<br>2. No	
	d) Not knowing costs for the intervention and hence jeopardising projects sustainability at the end of DP projects	1. Yes<br>2. No	
	e) Others (Mention)		
28.	What are more explanations for the responses in question 27		
	a)		
	b)		
	c)		
	d)		

PART B: GENERAL INFORMATION REGARDING ENGAGEMENT OF DPs IN DISTRICT CCHP DEVELOPMENT PROCESSES:

S/N	Question	Response	Code
9.	How do you involve development partners in CCHP planning of this District?		
	a) By engaging them in pre planning meeting	1. Yes<br>2.No	
	b) DPs submitting their annual plans and commitment to be included in the CCHP	1. Yes<br>2. No	
	c) Sharing health strategic plan to guide DPs prioritisation	1. Yes<br>2. No	
	d) Sharing with DPs the CCHP planning guideline and other national health guidelines to guide their planning	1. Yes<br>2. No	
	e) Others (Mention)		
10.	What are the guiding documents (policies, laws, guideline, protocol) on the participation, and integration of DP plans into the District CCHP?		
	a) Annual District CCHP	1. Yes<br>2. No	
	b) Annual District Health stakeholders meeting report	1. Yes<br>2. No	
	c) Annual Health Information (HIMS) report	1. Yes<br>2. No	
	d) District Planning and Reporting Tool	1. Yes<br>2. No	
	e)National joint assistance (aids) strategy framework of year 2006	1. Yes<br>2. No	

PART E: COORDINATION OF DPs ACTIVITIES

S/N	Question	Responses	Code
29.	Do you regularly receive action /work plans from DPs showing implementation schedule?	1. Yes 2. No	
30.	If yes to question 29 how often?		
	a) Monthly	1. Yes 2. No	
	b) quarterly	1. Yes 2. No	
	c) Biannually	1. Yes 2. No	
	d) Annually	1. Yes 2. No	
	e) Others (specify)_____________________________________		
31.	How does the health department coordinate implementation of partner plans /activities?		
	a) By holding meetings with DPs	1. Yes 2. No	
	b) Established communication network with DPs	1. Yes 2. No	
	c) Having a lead partner	1. Yes 2. No	
	d) Having DPs implementation schedule	1. Yes 2. No	

	e) Others (specify)___________________________________		
32.	Are there any District Strategies for enhancing engagement and integration of local development partner plans into CCHP?	1. Yes 2. No	
33.	If the answer is Yes to Question 32 above, share with us the strategies		
	a) Joint district coordination meeting	1. Yes 2. No	
	b) DPs networking	1. Yes 2. No	
	c) Having a lead partner	1. Yes 2. No	
	d) Having DPs implementation schedule	1. Yes 2. No	
	e) Joint supervision and follow up of DP implementation of the action plans	1. Yes 2. No	
	f) Others specify		
34.	Normally, what do you do to mitigate the effects of non-integrated partner plans?		
	a) Getting implementation schedule from the DPs	1. Yes 2. No	
	b) Establishing a district staff training plan for short training to minimize constant task shifting	1. Yes 2. No	
	c) DPs networking	1. Yes 2. No	
	d) Sharing District priorities to DPs	1. Yes 2.	

		No	
	e) Others specify		
35.	What is generally recommended in order to improve participation of local district development partners in CCHP Planning, implementation and monitoring processes?		
	a) Strengthen communication and DPs networking	1. Yes<br>2. No	
	b) Policies to strengthen compliance	1. Yes<br>2. No	
	c) Review the DP coordination	1. Yes<br>2. No	
	d) Others (Specify)		

Thank you for your participation
