# Supplementary material for: Operational challenges of engaging development partners in district health planning in Tanzania
Source: BMC Public Health. 2022 Jan 29;22:200. doi: 10.1186/s12889-022-12520-6 (PMC8800550; doi:10.1186/s12889-022-12520-6)
Supplement: Supplementary file 3 — Additional file 3. [file 12889_2022_12520_MOESM3_ESM.pdf]

Additional file 3: Development Partner Assessment Questionnaire

Questionnaire ID: [ ][ ]

Date of Interview: dd/mm/yyyy: [ ][ ] /[ ][ ] /[ ][ ] [ ][ ]

PART A: INTRODUCTION

<table>
<tr><th>S/N</th><th colspan="2">Question</th><th>Answer</th><th>Code</th></tr>
<tr><td>1.</td><td colspan="2">District name</td><td>1. Bahi 2. Kinondoni</td><td></td></tr>
<tr><td>2.</td><td colspan="2">Name of the interviewee (optional)</td><td colspan="2"></td></tr>
<tr><td>3.</td><td colspan="4">Organization name</td></tr>
<tr><td rowspan="2">4.</td><td>a) Type of Development Partner</td><td colspan="2">1.International NGO
2.National NGO
3.Regional NGO
4 District .NGO
5.Bilateral Project
6.Multi-lateral project
7. FBO (International/National)
8.FBO (Regional/District)
9.CBO
10.Other Mention__________</td><td></td></tr>
<tr><td colspan="4">b) Position of the interviewee in the organisation____</td></tr>
</table>

	c) How long have you been working with this organisation? [ ] [ ]		
5.	Duration of DP operation in the District in years [ ][ ] [ ]		
6.	Does your organisation have Memorandum Of Understanding with this District?	1.Yes 2.No	
7.	If Yes to question 6, what is the current duration of MOU under implementation?(Years) [ ] [ ]		
8.	Nature of partner support(please explain the interventions you support)		
	a) Capacity building to the management (CHMT and Facility governing teams/committees)	1.Yes 2.No	
	b) Capacity building to service providers including trainings, supervision and mentorship,	1.Yes 2.No	
	c) Supporting community interventions (Community health funds)	1.Yes 2.No	
	d) Supporting disease specific interventions such as HIV ,TB or Malaria interventions	1.Yes 2.No	
	e) Supporting water hygiene and sanitation interventions.	1.Yes 2.No	
	f) Nutrition intervention	1.Yes 2.No	
	g) Reproductive and child health services	1.Yes 2.No	
	h) Others (please specify)		

PART B: INFORMATION REGARDING DEVELOPMENT PARTNER PLAN:

S/N	Question	Answer	Response
9.	In preparing your organisation plan, do you use any document/reference from the district??	1.Yes<br>2.No	
10.	If the answer to question 9 above is yes, Which documents from the District did you use in developing your organisation plan?		
	a) The annual district health plan (CCHP)	1.Yes<br>2.No	
	b) District stakeholders meeting report	1.Yes<br>2.No	
	c) District HIMS (MTUHA) report	1.Yes<br>2.No	
	d) District Planning and Reporting Tool	1.Yes<br>2.No	
	e) National joint assistance (aids) strategy framework of year 2006	1.Yes<br>2.No	
	f) Others (please specify)	1.Yes<br>2.No	
11.	Does the District have a copy of your this year annual plan?	1.Yes<br>2.No	

12.	If the answer to question 11 above is NO, Please explain		
	a) Did not have enough copies	1.Yes<br>2.No	
	b) Didn't see the relevance /not demanded	1.Yes<br>2.No	
	c) Not requested from the district	1.Yes<br>2.No	
	d) Others (please specify)		
13.	Do you have a copy of the CCHP of this District for the FY 2013/14	1.Yes<br>2.No	
14.	What are the planning processes used in developing your organisation plan?		
	a) Doing the need assessment/feasibility study	1.Yes<br>2.No	
	b) Getting information on the local priorities	1.Yes<br>2.No	
	c) Goal setting	1.Yes<br>2.No	
	d) setting financing and implementation strategies	1.Yes<br>2.No	
	e) Others (please explain)		
17.	a) Is it possible to tell us your organisation	1.Yes	

	budget for the FY 2013/14, that is, how much did your organisation plan to spend in this District (excluding the organisation administrative costs)?	2.No	
	b) If yes to question 17a how much was budgeted (Tshs)		
	c) If no to Question 17a, what are the reasons?	1. Not sure of the budget<br>2. Is a confidential information	
	d) If yes to question 17a, how much has been released to date (Tshs)?	1. More 75%<br>2. 50 %<br>3.Less than 25%<br>4.Not at all<br>5.I Don't know	
18.	In implementing your plans, do you share your implementation action plan (schedule) with the District?	1.Yes<br>2.No	
19.	.If yes, how often?		
	a) Once a year	1.Yes	

		2.No	
	b) Every six months	1.Yes<br>2.No	
	c) Quarterly	1.Yes<br>2.No	
	d) Monthly	1.Yes<br>2.No	
	e) Weekly	1.Yes<br>2.No	

PART C: INFORMATION REGARDING ENGAGEMENT AND INTERGRATION OF THE ORGANISATION PLAN INTO DISTRICT CCHP

S/N	Question	Answer	Code
20.	Did your organisation participate in the 2014/15 CCHP council pre planning meeting?	1.Yes 2.No	
21.	Did you submit your organisation plan with CHPT to be integrated in the CCHP for the year 2013/14 in writings?	1.Yes 2.No	
22.	If yes to Question 21, please check the number of organisation activities submitted to district CHPT and submission letter		
23.	Check number of activities included in the plan if copy of CCHP is		

	available or feedback letter from CHMT	
24.	How has your organisation benefited from the processes of developing the CCHP?	
	a) Reducing organisation implementation expenses	1.Yes 2.No	
	b) Easy to get areas to focus on (getting priorities from the locals)	1.Yes 2.No	
	c) Easy to link and cooperate with other partners	1.Yes 2.No	
	d) Achieve organisation visibility	1.Yes 2.No	
	e) No benefits	1.Yes 2.No	
	f) Others (please specify)	

PART D: PERCEIVED LEVEL OF THE ORGANISATION PARTICIPATION/ENGAGEMENT IN DISTRICT CCHP:

**S/N**	**Question**	**Level of participation/engagement**<br>1. ZERO Participation<br>2. Minimum /Partial Participation<br>3. Substantial /Full Participation

25.	Generally, what is the level of participation/engagement of your organisation in the processes of developing this district CCHP for the FY 2014/15?	
26.	What is the level of participation of your organisation in the following CCHP Planning Processes:	
	a) Identifying priority health problems /intervention to be addressed in the 2014/15 CCHP Plan?	
	b) Allocating resources to the interventions?	
	c) Developing CCHP Action Plan?	
	d) Developing the capacity of the Council Health Planning Team?	
	e) Implementation of CCHP activities?	
	f) Quarterly review of the CCHP implementation?	

PART E: DP PERCEPTION OF THE DISTRICT HEALTH PLANNING PROCESSES:

S/N	Question	Answer	Code
27.	How effective is the District /Government Commitment in involving the development partners	1.Very Effective<br>2.Moderate	

	in developing CCHP in this District?	effective<br>3.Low<br>4.Not effective	
28.	Please explain		
	a)		
	b)		
	c)		
29.	Does the current CCHP reflect views /needs of the local district development partners?	1.Yes<br>2.No	
30.	If yes to question number 29 above, please explain		
	a) DPs were engaged in preparation of CCHP and able to link with other partners for more collaboration	1.Yes<br>2.No	
	b) We got feedback on the activities which were able to be included in the CCHP and note the areas for further investments	1.Yes<br>2.No	
	c) DPs participate in evaluation of the implementation of the CCHP	1.Yes<br>2.No	
	d) It was possible to note the volume of contribution made by our organisation through CCHP	1.Yes<br>2.No	

	e) Others (please explain)		
31.	What do you think are the reasons for some district donor partners not to integrate their organisation plans into the CCHP?		
	a) Not knowing the government guidelines	1.Yes<br>2.No	
	b) Not in the policy of our prime donor	1.Yes<br>2.No	
	c) Increases projects costs	1.Yes<br>2.No	
	d) Not necessary that the government recognises contribution of the partner	1.Yes<br>2.No	
	e) Others (please explain)		
32.	What do you think are the reasons for some of the district DPs to integrate plans into the CCHP?		
	a) Adhere to government policies, guidelines and directives	1.Yes<br>2.No	
	b) It is the policy of their donor	1.Yes<br>2.No	
	c) Lower operational/implementation expenses	1.Yes<br>2.No	
	d) To get recognition of their contribution	1.Yes	

		2.No	
	e) Others (please explain)		
33.	Is your organisation plan integrated in the district CCHP for the FY 2014/15?	1.Yes<br>2.No	
34.	What are the benefits of integrating DP plans into the CCHP?		
	a) Reduce operational costs	1.Yes<br>2.No	
	b) Improve organization visibility	1.Yes<br>2.No	
	c) Reduce staffs workload	1.Yes<br>2.No	
	d) Getting harmonized reports	1.Yes<br>2.No	
	e) Easy to get district health actual costs.	1.Yes<br>2.No	
	e) Others please explain		
35.	How are the district development partners affected by non-integration of the donor plans into the CCHP?		
	a)		
	b)		

	c)		
	d)		
	e)		
36.	In your opinion, is it possible for all partner plans to be integrated into the CCHP?	1. Yes 2. No	
37.	If Yes to question 36 above, please explain		
	a)		
	b)		
	c)		
38.	What is generally recommended to change in order to improve the participation and integration of donor partner plans into the CCHP and its impacts?		
	a)		
	b)		
	c)		
	d)		

THANK YOU FOR YOUR PARTICIPATION
