# Supplementary material for: Operational challenges of engaging development partners in district health planning in Tanzania
Source: BMC Public Health. 2022 Jan 29;22:200. doi: 10.1186/s12889-022-12520-6 (PMC8800550; doi:10.1186/s12889-022-12520-6)
Supplement: Supplementary file 4 — Additional file 4. [file 12889_2022_12520_MOESM4_ESM.pdf]

**Additional file 4a. In-depth Interview Guide For Council Health Planning Team members**

**<u>Instructions for the Interviewer</u>**

**Step 1: <u>Informed Consent</u>:** *Ask the participant for a few minutes of their time. Introduce yourself and the study. Begin by asking for informed consent from the participant as per the training. If consent is granted, let the participant sign it and leave the informed consent sheet with the participant.*

**Was verbal informed consent obtained for each participant before the start of the IDI?**

**YES ________ (proceed with IDI)**

**NO ________ (STOP! Thank the participants for their time.**

***<u>Interviewer</u>:*** *Read the following statement. .*

*"I will ask you a series of questions about your* ***experience and challenges regarding integration of donor partner plans into the district plans****. I will ask you questions and will write down your responses to the questions. I will also record the discussions to be sure that we capture your responses. I expect this discussion to last approximately one hour. There are no right or wrong answers and your participation will have no harm to you or your institution. Please feel free to ask me if you have any questions or if you do not understand any of my questions. You are free to end your participation at any time or to remain quiet when I ask any questions that you do not wish to answer."*

**Step 2***: Please complete the basic demographic table for the participant as they sign in. This form will have an ID number for each participant.*

**Step 3:** *Proceed to the IDI. Start by reiterating the importance of confidentiality. As you ask the questions, please probe to obtain as many responses as possible for each question.*

**NOTE TO FACILITATOR:** Most questions below have probe or follow-up questions to fuel your discussion with participants. If participants have already addressed the questions asked in the probes, then move onto the next question. Please feel free to explore a response given by a participant if it is unusual or interesting.

**Facilitator ID** _________ **Note-taker ID:** ________________

1. **IDI Date (DD/MM/YYYY)** __________________
2. **District #:** ______________
3. **Time Start** _______ Time **Finish** ___________

**Step 4.Demographics of IDI Participants**

| ID | SEX | Age | Level of Education | Participant type<br>1) DMO<br>2) DHS<br>3) DCDO<br>4)DRCHCO | Experience with District planning<br>1) < 2 yrs.<br>2) >2 yrs. |
|---|---|---|---|---|---|
| A | | | | | |

**Step 5.THEMES FOR DISCUSSION**

**THEME 1: Level of participation and integration of donor plans into the District health plan**

1.1 Tell me about your experience in the participation of District donor partners in the preparation of District comprehensive health planning processes.

1.2 Do donor partners normally participate in the district health planning?

**Probe Questions**:

a. If they do participate; can you tell how, do they present their plans in writing to be integrated into the CCHP? To what extent?
b. If no, what prevents donor partners from participating in the District planning process?

c. What are the guiding documents (policies, guidelines, protocols for the integration of plans?
d. How well are the documents known to both district planning team and the donors?
e. What are the Mechanisms for ensuring compliance of donors into those policies, guidelines for integration of their activities into the CCHP?

1.3 Can you explain if there is any situation that restricts the district from involving all district health donors in the preparation processes of the CCHP?

**Probe Questions**:

a. If there is financial (cost) issue?
b. Time ”
c. Any planrep tool issue
d. Coordination among donor themselves

**THEME 2: Causes of Non-Integration**

2.1 Can you explain if there is any situation that restricts the district from fully integrating donor plans into the CCHP?

**Probe Questions**:

e. If there are any restrictive policies?
f. Any CHPT knowledge, capacity issue”
g. Any planrep tool issue, does it allow all donors plans to be integrated
h. Any management coordination and control issue

**THEME 3:Effect of Non-Integration**

3.1 What are the effects of non-integrated donor plans to the functioning of the CHMT and health facility?

3.2. How are the donor organizations affected if they do not integrate their activities into the CCHP?

**THEME 4: Health Donor Partner Coordination Mechanisms at the District**

4.1 What are the currently available district mechanisms for coordinating district donor activities implementation?

**Probe Questions**:

a. What are the gaps in these mechanisms?

**THEME 5: Strategies to Improve partner Coordination at the District**

5.1 What can be done to improve district health donor activities coordinating mechanisms?

**Additional file 4b: In-depth Interview Guide for Development Partners**

**Instructions for the Interviewer**

**Step 1: Informed Consent:** *Ask the participant for a few minutes of their time. Introduce yourself and the study. Begin by asking for informed consent from the participant as per the training.. If consent is granted, let the participant sign it and leave the informed consent sheet with the participant.*

**Was verbal informed consent obtained for each participant before the start of the IDI?**

**YES ________ (proceed with IDI)**

**NO ________ (STOP! Thank the participants for their time.**

***Interviewer:*** *Read the following statement. .*

*"I will ask you a series of questions about your* ***experience and challenges regarding integration of donor partner plans into the district plans****. I will ask you questions and will write down your responses to the questions. I will also record the discussions to be sure that we capture your responses. I expect this discussion to last approximately one hour. There are no right or wrong answers and your participation will have no harm to you or your institution. Please feel free to ask me if you have any questions or if you do not understand any of my questions. You are free to end your participation at any time or to remain quiet when I ask any questions that you do not wish to answer."*

**Step 2***: Please complete the basic demographic table for the participant as they sign in. This form will have an ID number for each participant.*

**Step 3:** *Proceed to the IDI. Start by reiterating the importance of confidentiality. As you ask the questions, please probe to obtain as many responses as possible for each question.*

**NOTE TO FACILITATOR:** Most questions below have probe or follow-up questions to fuel your discussion with participants. If participants have already addressed the questions asked in the probes then move onto the next question. Please feel free to explore a response given by a participant if it is unusual or interesting.

**Facilitator ID** _________ **Note-taker ID:** _________________

4. **IDI Date (DD/MM/YYYY)** __________________
5. **District #:** ______________
6. **Time Start** _______ Time **Finish** ___________

**Step 4.Demographics of IDI Participants**

| **ID** | **SEX** | **Age** | **Level of Education** | **Participant type**<br>NGO<br>FBO<br>Donor Project<br>Philanthropy | **Experience with District planning**<br>**3) < 2 yrs.**<br>**4) >2 yrs.** |
|---|---|---|---|---|---|
| **A** | | | | | |

**Step 5.THEMES FOR DISCUSSION**

**<u>THEME 1: Level of participation and integration of donor plans into the District health plan</u>**

1.1 Tell me about your experience of the participation of your organization in the preparation of District comprehensive health planning processes

1.4 Do district donor partners normally participate in the district health planning? Or is your organization willing to share its plans with the district government?

**Probe Questions**:

f. If they do participate; can you tell how, do they present their plans in writing to DMO to be integrated into the CCHP? To what extent? How are they coordinated? Does the NGO representative take views and plans from all donors/NGO for representation?

g. If no, what prevents donor partners from participating in the District planning process?
h. What are the guiding documents (policies, guidelines, protocols for the integration of plans?
i. How well are the documents known to both district planning team and the donors?
j. What are the mechanisms for ensuring compliance of donors into those policies, guidelines for integration of their activities into the CCHP and how are they implemented?

1.5 Can you explain if there is any situation that restricts the district from involving all district health donors in the preparation processes of the CCHP?

**Probe Questions**:

i. If there is financial (cost) issue?
j. Time ”
k. Any planrep tool issue
l. Coordination among donor themselves

**<u>THEME 2: Causes of Non-Integration</u>**

2.1 Can you explain if there is any situation that restricts the district donors from fully integrating donor plans into the CCHP?

**Probe Questions**:

m. If there are any restrictive policies?
n. Any CHPT knowledge, capacity issue”
o. Any planrep tool issue, does it allow all donors plans to be integrated
p. Any management coordination and control issue

**THEME 3:Effect of Non-Integration**

3.1 What are the effects of non-integrated donor plans to the functioning of the CHMT and health facility?

3.2. How will the donor organizations be affected if they do not integrate their activities into the CCHP?

**THEME 4: Health Donor Partner Coordination Mechanisms at the District**

4.2 What are the currently available district mechanisms for coordinating district donor activities implementation?

**Probe Questions**:

b. What are the gaps in these mechanisms?

**THEME 5: Strategies to Improve partner Coordination at the District**

5.1 What can be done to improve district health donor activities coordinating mechanisms?
